# METABOLIC SYNDROME, GLUCOSE INTOLERANCE, AND PHYSICAL RESILIENCE

**DOI:** 10.1093/geroni/igac059.008

**Published:** 2022-12-20

**Authors:** Rita Kalyani, Ravi Varadhan, Qian-Li Xue, Frederick Sieber, Philip Imus, Brian Buta, Karen Bandeen-Roche, Jeremy Walston

**Affiliations:** Johns Hopkins University School of Medicine, Baltimore, Maryland, United States; Johns Hopkins University School of Medicine, Baltimore, Maryland, United States; Johns Hopkins University, Baltimore, Maryland, United States; Johns Hopkins University School of Medicine, Baltimore, Maryland, United States; Johns Hopkins University School of Medicine, Baltimore, Maryland, United States; Johns Hopkins University School of Medicine, Baltimore, Maryland, United States; Johns Hopkins Bloomberg School of Public Health, Baltimore, Maryland, United States; Johns Hopkins University, Baltimore, Maryland, United States

## Abstract

When confronted with a major physical stressor, some older adults are able to recover to baseline with minimal changes, while others undergo irreversible declines. The ability to adapt and recover well from physical stress is called physical resilience and may be impeded by altered glucose-insulin dynamics. Among 55 participants in the pilot phase of the Study of Physical Resilience IN Geriatrics(SPRING), we examined associations between metabolic syndrome and/or diabetes status to physical resiliency recovery parameters after the clinical stressors total knee replacement and bone marrow transplant. These parameters included health outcomes, functional recovery, and pain in the weeks after the intervention. Additionally, we hypothesized that less resilient individuals would have abnormal glucose and insulin responses to a 75-gram oral glucose tolerance test at 0, 30, 60, 120 minutes and tested this in a subset (n=40) without known diabetes. Exploratory analyses of this pilot study will be presented during this session.

